# Sling Exercise for Chronic Low Back Pain: A Systematic Review and Meta-Analysis

**DOI:** 10.1371/journal.pone.0099307

**Published:** 2014-06-11

**Authors:** Yu-Shan Yue, Xu-Dong Wang, Bin Xie, Zhong-Han Li, Bing-Lin Chen, Xue-Qiang Wang, Yi Zhu

**Affiliations:** 1 Second School of Clinical Medical, Nanjing University of Chinese Medicine, Nanjing, Jiangsu, China; 2 Institute of Literature in Chinese Medicine, Nanjing University of Chinese Medicine, Nanjing, Jiangsu, China; 3 First School of Clinical Medical, Nanjing University of Chinese Medicine, Nanjing, Jiangsu, China; 4 Applied Health Science Department, University of Waterloo, Waterloo, Ontario, Canada; 5 Department of Sport Rehabilitation, Shanghai University of Sport, Shanghai, China; University Hospital Basel, Switzerland

## Abstract

**Background:**

Trials on sling exercise (SE), commonly performed to manage chronic low back pain (LBP), yield conflicting results. This study aimed to review the effects of SE on chronic LBP.

**Methods:**

The randomized controlled trials comparing SE with other treatments or no treatment, published up to August 2013, were identified by electronic searches. Primary outcomes were pain, function, and return to work. The weighted mean difference (WMD) and 95% confidence interval (CI) were calculated, using a random-effects model.

**Results:**

Risk of bias was rated as high in 9 included trials, where some important quality components such as blinding were absent and sample sizes were generally small. We found no clinically relevant differences in pain or function between SE and other forms of exercise, traditional Chinese medical therapy, or in addition to acupuncture. Based on two trials, SE was more effective than thermomagnetic therapy at reducing pain (short-term: WMD –13.90, 95% CI –22.19 to –5.62; long-term: WMD –26.20, 95% CI –31.32 to –21.08) and improving function (short-term: WMD –10.54, 95% CI –14.32 to –6.75; long-term: WMD –25.75, 95% CI –30.79 to –20.71). In one trial we found statistically significant differences between SE and physical agents combined with drug therapy (meloxicam combined with eperisone hydrochloride) but of borderline clinical relevance for pain (short-term: WMD –15.00, 95% CI –19.64 to −10.36) and function (short-term: WMD −10.00; 95% CI −13.70 to −6.30). There was substantial heterogeneity among the two trials comparing SE and thermomagnetic therapy; both these trials and the trial comparing SE with physical agents combined with drug therapy had serious methodological limitations.

**Interpretation:**

Based on limited evidence from 2 trials, SE was more effective for LBP than thermomagnetic therapy. Clinically relevant differences in effects between SE and other forms of exercise, physical agents combined with drug therapy, traditional Chinese medical therapy, or in addition to acupuncture could not be found. More high-quality randomized trials on the topic are warranted.

## Introduction

Low back pain (LBP) is a very common disorder [1], with approximately 84% of adults experiencing an episode of LBP at some point during their lifetimes [2] and variable recurrence rates (5% to 60%) [3]. LBP, identified as the leading disability contributor [4], may result in a reduced level of physical capacity [5]–[9], negative psychological effects [10]–[17], and reduction in the quality of life; as such, LBP is one of the most common reasons that patients opt to undergo health care [18], [19]. In the United States, back pain-related lost productive work time in workers aged between 40 and 65 years costs employers an estimated $7.40 billion per year [20]; and an estimated $50 billion is spent annually on LBP treatments [21]. In Australia, direct medical costs associated with LBP treatments are estimated at more than $1 billion per year, with additional $8 billion covering indirect expenses [22]. Although the outcomes for many individuals with first-episode LBP are positive, 20% of these cases may develop into chronic LBP, which is defined as a chronic condition of LBP lasting for at least three months or longer [3], [23]–[26]. And chronic LBP accounts for three-quarters of the total direct and indirect costs of medical care and lost productivity associated with LBP [27]. Hence, it is essential to improve the efficiency of treatment of chronic LBP.

In clinical guidelines, exercise therapy is considered as an effective treatment to reduce self-reported pain and improve the back pain specific functional status of participants with chronic LBP [28]. Sling exercise (SE) is a specific form of exercise established by Meier [29] to rehabilitate professional German sportsmen and later developed by Kirkesola to treat motor problems [30]. SE supports or suspends the pelvis and lower extremities in a sling, and allows an individual to use his or her body weight to provide resistance [31], [32]. This exercise minimizes the use of global muscles without pain as local muscles are activated. This procedure can be performed easily by using a sling and an elastic cord to offset body weight; however, this procedure is difficult when an unstable surface is used [31]. SE has been claimed to reduce pain, normalize muscle response patterns, retrain muscle motor units, re-operate inhibited actions, and improve damaged postural adjustment abilities [33].

Published randomized controlled trials (RCTs) have assessed the effects of SE on the treatment of chronic LBP [34]–[42]. However, RCTs that assess the effects of SE compared with other forms of exercise on self-reported pain in patients with chronic LBP have presented conflicting findings [34], [35], [38], [41]. In some of these studies, no differences between SE and other forms of exercise have been found [34], [41]. In other studies, SE exhibits more advantages than other forms of exercise [35], [38]. Studies examining back pain specific functional status between SE and other forms of exercise have also yielded inconsistent results [34], [36], [38]. Some studies have found no differences between patients receiving SE and other forms of exercise [34], [38]. The remaining trial has demonstrated an increased improvement in patients receiving SE [36]. In two randomized trials, in which SE is compared with traditional Chinese medical therapies [37], [42], one trial has shown that pain is reduced when SE is used [37]; the other trial has demonstrated that SE produced less pain reduction compared with a specific traditional Chinese medical therapy [42]. To the best of our knowledge, no published systematic review or guideline on the efficacy of SE for subjects with chronic LBP has been conducted. Therefore, the effectiveness of SE compared with other treatments remains unclear. Clinicians may also be undecided whether or not SE should be prescribed in patients with chronic LBP. It is necessary to conduct a systematic evaluation of RCTs focusing on SE for patients with chronic LBP in this case.

We performed a systematic review to assess the efficacy of SE in participants with chronic LBP. The results of this study provided information to help clinicians come up with evidence-based decisions on the use of SE for patients with chronic LBP.

## Methods

### Literature Search

The following databases were searched from the earliest available date to August 2013: Cochrane Library; Cumulative Index to Nursing and Allied Health Literature; Pubmed; the Web of Science; Embase; the Physiotherapy Evidence Database; Chinese Biomedical Literature Database; Wanfang Database; and China National Knowledge Infrastructure. We used “LBP”, “SE”, and “RCTs” as search terms (full details of the search in File S1). To increase the chance of finding all relevant publications describing the effects of SE on LBP, we did not set limitations on language, year, or status during the initial search.

To identify gray literature, we contacted experts and inquired regarding materials not listed in these databases. The International Controlled Trials Registry Platform was also searched, in which “SE” and “LBP” were used as key words, to obtain relevant registered trials, which may contain additional data, but did not have any published papers. The reference lists of identified articles were screened manually for additional studies.

### Inclusion Criteria

#### Types of studies

Only RCTs investigating the use of SE as treatment for chronic LBP were included. No language or publication date restrictions were applied.

#### Types of participants

The study samples included patients with chronic LBP affected for longer than three months. Unless an adequate washout period was described, patients who were exposed to similar treatments prior to the study were not included.

#### Types of interventions

We included articles in which SE was compared with no or placebo treatment, as well as any other treatment for chronic LBP. In the SE training program, the neuromuscular activation was induced to regain normal functional movement patterns in patients with musculoskeletal disorders [33]. SE was performed using specially designed devices, such as Record Trainer or TerapiMaster [34], [43]. Trials in which a treatment was applied with concomitant therapy were accepted as long as similar treatments were applied in the control conditions.

#### Types of outcome measures

The findings were analyzed in three primary outcome categories: (1) self-reported pain; (2) back pain specific functional status; and (3) return to work (expressed as the number of days of sick leave or the proportion of patients returned to work). Secondary outcomes of this review include global improvement, health-related quality of life, satisfaction with treatment, and adverse events. We categorized outcomes as long term (≥12 months), intermediate (closer to 6 months), or short term (post-treatment assessment no longer than 12 weeks).

### Selection of Studies

Two reviewers (Yue YS, Zhu Y) independently screened for potentially relevant titles and abstracts based on the pre-specified criteria; full-text articles were retrieved whenever necessary. Any disagreements were resolved by discussion or consultation with a third independent reviewer (Wang XQ) if necessary.

### Data Extraction

Two independent reviewers (Yue YS, Zhu Y) abstracted and cross-checked the data obtained from the included trials. These data were then compiled in a pre-designed data extraction form. The data extraction form included study design (treatment allocation, concealed allocation, blinding, intention-to-treat analysis, etc.), participant characteristics (mean age, group distribution, etc.), description of control and treatment interventions (SE style and practices, type of control intervention, frequency, total duration, etc.), outcome measures used, and follow-up period. Any disagreements were resolved by discussion to obtain a consensus. The authors were also contacted to provide further information when deemed necessary.

### Quality Assessment

The two independent reviewers (Yue YS, Zhu Y) used the Cochrane Collaboration’s risk of bias tool to evaluate the methodological quality of all included studies. The following domains were evaluated: random sequence generation, allocation concealment, blinding of participants and personnel, blinding of outcome assessments, incomplete outcome data, selective reporting, and other bias [44]. For each domain, each study’s description of methods was examined and the judgment regarding potential bias was made, according to three categories: low risk, high risk and unclear risk [45]. The overall risk of bias of individual study was rated as low (low risk of bias for all domains), high (high risk of bias for one or more domains) or unclear (unclear risk of bias for one or more domains) [46]. A third independent reviewer (Wang XQ) was consulted to resolve disagreements.

### Statistical Analysis

Data were analyzed with the Review Manager statistical software (RevMan version 5.2) by using a random-effects model. Random effects meta-analysis was performed to combine the results because we predicted that study characteristics and other factors were different, suggesting that the effects may differ across studies. Chi-square test was performed to detect statistically significant heterogeneity [47]. We then estimated the amount of heterogeneity among studies by using the *I^2^* statistic: <25%, low; <50%, moderate heterogeneity; and >50%, substantial heterogeneity [48]. Heterogeneity was further investigated by checking data extracted from outlier studies and exploring the effects of study exclusion in sensitivity analyses. No funnel plots or assessments for publication bias were performed because of the small number of trials (maximum five trials) that were pooled in the comparisons included in this literature.

The control conditions were divided into five groups: other forms of exercise; traditional Chinese medical therapy; thermomagnetic therapy; physical agents in combination with drug therapy; and no treatment. We then performed separate meta-analyses for short-term, intermediate, and long-term follow-up time points. One study included two different control groups, which were individually considered during analysis [49].

Analyses were only possible for self-reported pain and back pain specific functional status because of absence of data for return to work, global improvement, health-related quality of life, satisfaction with treatment, and adverse events. In the included studies, several outcome measures were used to assess the constructs of self-reported pain, including a 10 mm or 100 mm visual analogue scale (VAS) [43] or 0-point to 10-point numerical pain rating scale (NPRS) [34], and the back pain specific functional status, such as 100-point Oswestry disability index (ODI) [40] or modified Oswestry disability index (M-ODI) [34]. Moderate to high correlations between the different measures of the two constructs were obtained [50]. In this literature, the individual trial outcomes for self-reported pain and back pain specific functional status were linearly rescaled to a 0 to 100 scoring system, in which lower scores indicated better outcomes, to facilitate the comparison and interpretability of the syntheses [49], [51], [52]. For example, we rescaled a NPRS pain score of 2.3±1.1 points (mean ± SD) out of 10 points to 23±11 points out of 100 points, where positive mean effect sizes indicated an improvement that is decreased pain. All of the variables included in the analysis were continuous; as such, the inverse variance method was used to calculate the weighted mean differences (WMDs) and the corresponding 95% confidence intervals (95% CIs) for each study based on the post-intervention means of each group and the pooled standard deviation. Two-sided statistical tests were performed. P<0.05 or 95% confidence interval that excluded a null result was considered statistically significant [53]. On the basis of current research on minimal clinically significant differences, a 20-point (of 100 points) improvement in pain [54] and a 10-point (of 100 points) improvement in functional outcomes [55] were considered as clinically meaningful.

## Results

### Study Selection

**Figure 1:**
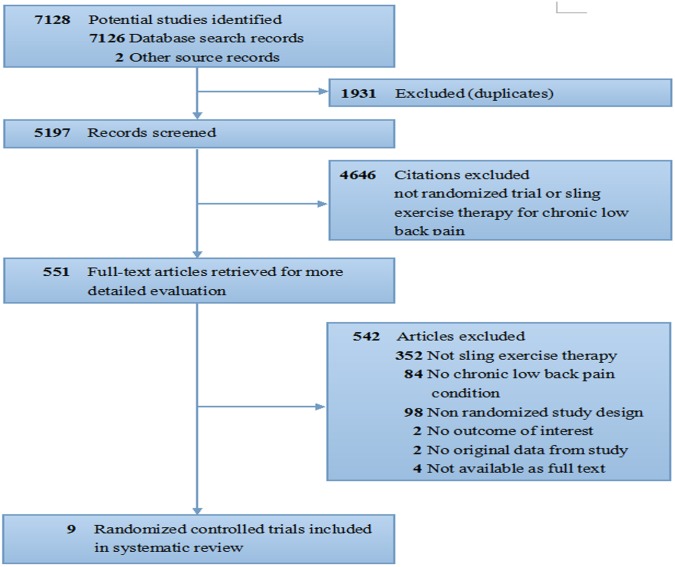
Review flow diagram.

Our initial search yielded 7,128 records. Among these data, 5,197 were retained after duplicates were removed. A total of 4,646 articles were excluded because these studies did not use randomized controlled trial designs, the recruited sample populations comprised patients without chronic LBP, or no adequate intervention group was used. The 551 remaining articles were assessed in detail for eligibility. Among these remaining articles, 542 were excluded. A total of 450 articles were non-RCTs or studies of unrelated intervention conditions. A total of 86 studies used participants without chronic LBP or reported on results not included in the predefined outcomes. Six articles [56]–[61] were not available for full text [56], [59]–[61] or had incomplete outcome data [57], [58], and the authors of these studies [56]–[61] did not respond to our repeated contacts. Thus, nine RCTs [34]–[42] with a total of 706 participants were included in this study. Figure 1 shows the flow chart from initial results of publication searches to final inclusion or exclusion.

### Description of Included Studies

We included 9 single center randomized controlled trials [34]–[42] aiming to examine the efficacy of SE on chronic LBP. Of these, one trial [34] was conducted in a primary care setting in Norway, seven trials [36]–[42] were done in Chinese hospitals, and one trial [35] was from Republic of Korea. Trials [34]–[42] were published between 2008 and 2012, and all [34]–[42] but two [34], [35] were published in Chinese language. The summarized characteristics of the studies [34]–[42] included in our systematic review are presented in Table 1.

**Table 1:** Characteristics of trials included in systematic review.

Article,Year	Patients Characteristic,Sample Size	Intervention	Duration oftrial period	Outcomes	Timepoint
Gao Baolong (2008)	Source: hospital 29patients (G1 = 15, G2 = 14);Mean age (SD): G1 = 37.0y(4.28), G2 = 35.0y (3.99).	G1: SE;G2: Massage.	five times aweek for 8 weeks.	Self-reported pain(VAS 0–10); Skeletal musclemetabolism (Serum CK,Serum LDH).	8 weeks.
Unsgaard-Tondel (2010)	Source: primary care andhospital 109 patients(G1 = 36, G2 = 36, G3 = 37);Mean age (SD): G1 = 43.4y(10.2), G2 = 40.9y (11.5),G3 = 36.0y (10.3).	G1: SE; G2: Motor controlexercise;G3: General exercise.	once a weekfor 8 weeks.	Self-reported pain(NPRS 0–10); Back painspecific functional status(M-ODI); Fear of physicalactivity and work (FABQ);Trunk flexion (FTF); Health careutilization (Low back pain therapy,Low back pain medication).	8 weeks; 14months.
Guo Xianfeng (2010)	Source: hospital 246patients (G1 = 82, G2 = 82,G3 = 82); Mean age (SD):G1 = 34.1y (6.5), G2 = 33.3y(6.5), G3 = 33.5y (5.0).	G1: Intensive therapy: Backschool+SE+Health ballexercise; G2: Home exercise:Back school+Aerobictraining+Freestandingexercise; G3: Conventional therapy:Back school+Physicalagents+Drug therapy.	SE: three times a weekfor the first8 weeks; Health ball exercise:fourtimes a week for the last4 weeks; Aerobic training:three times a week for12 weeks; Freestandingexercise: four times aweek for 12 weeks; Physicalagents: five times a week for thefirst 4 weeks; Drug therapy:Meloxicam - once a day for thefirst 4 weeks, Eperisonehydrochloride tablet - three timesa day for the first 4 weeks.	Self-reported pain(NPRS 0–10); Back painspecific functionalstatus (M-ODI).	4 weeks; 12weeks; 24weeks.
Qin Jiang (2010)	Source: hospital 34patients(G1 = 12, G2 = 12, G3 = 10);Mean age (SD): 49.94y(11.90).	G1: SE; G2: SE+Magnetic fieldwarmer vibration synthetictherapy; G3: Immobilized withwaistline+Williamsgymnastics training.	SE: once every two days for 6weeks; Magnetic field warmervibration synthetic therapy: onceevery two days for 6 weeks;Williams gymnastics training:three times a day for 6 weeks.	Self-reported pain (NPRS0–10); Back pain specificfunctional status (ODI).	6 weeks; 18weeks.
Jin Miao (2011)	Source: hospital 60patients (G1 = 20, G2 = 20,G3 = 20); Mean age (SD):G1 = 45.97y(2.68), G2 = 44.74y (2.54),G3 = 44.79y (2.80).	G1: Intermediate frequency electrotherapy+SE; G2: Intermediate frequency electrotherapy+Thermomagnetic therapy; G3: Drug therapy.	Intermediate frequencyelectrotherapy: once a day for aweek; SE: once a dayfor a week; Thermomagnetictherapy: once a day for a week;Drug therapy: Gentongpingcapsule - two times a day for aweek, Mecobalamin- three times a day for a week.	Clinical effect (Criteria ofdiagnostic efficacy onsyndrome of traditionalChinese medicine); Self-reported pain (VAS 0–10);Anxiety state (HAMD);Ability of daily life(ADLRS); Back painspecific functional status(ODI); Joint function(ROM).	1 week; 5weeks.
Hu Yuan (2011)	Source: hospital 100patients (G1 = 50, G2 = 50);Mean age (SD):G1 = 40.39y (10.68),G2 = 39.13y(9.38)[based oncompleted cases].	G1: Back school+SE;G2: Backschool+Thermomagnetictherapy.	three times aweek for 6 weeks.	Self-reported pain (VAS0–10); Back pain specificfunctional status (ODI).	6 weeks; 13.5months.
Yoo (2012)	Source: university 30patients (G1 = 15, G2 = 15);Mean age (SD): G1 = 20.1y(0.7), G2 = 20.5y (0.5).	G1: SE;G2: Mat exercise.	three times aweek for 4 weeks.	Self-reported pain(VAS 0–10); Trunkextensor strength(The Tergumed Device).	4 weeks.
Wang Cong (2012)	Source: hospital 38patients (G1 = 19, G2 = 19);Mean age(SD): G1 = 36.95y (11.78),G2 = 36.05y (10.80).	G1: Back school+SE;G2: Back school+Freestandingexercise+Health ball training.	three times aweek for 8 weeks.	Self-reported pain (VAS0–10); Back pain specificfunctional status (ODI[exclude sexual life item]).	8 weeks; 12weeks; 20weeks.
Liu Pan (2012)	Source: community andhospital 60 patients(G1 = 20, G2 = 20, G3 = 20);Mean age (SD):G1 = 46.73y(11.58), G2 = 44.60y(10.57), G3 = 43.28y(10.34).	G1: SE; G2: Acupuncturetherapy; G3: Acupuncturetherapy+SE.	seven times aweek for 4 weeks.	Self-reported pain (VAS 0–10); Disability (JOA).	the first day; 2 weeks; 4 weeks.

**Abbreviations** G: Group; SD: Standard deviation; SE: Sling exercise; VAS: Visual analog scale; CK: Creatine kinase; LDH: Lactate dehydrogenase; NPRS: Numeric pain rating scale; M-ODI: Modified oswestry disability index; FABQ: Fear-avoidance beliefs questionnaire; FTF: the Fingertip-to-Floor test (in centimeters); ODI: Oswestry disability index; HAMD: Hamilton rating scale for depression; ADLRS: Activity of daily living rating scale; ROM: Range of motion; JOA: Japanese Orthopedic Association scores for assessment of low back pain.

#### Participants

Data from a total of 706 participants (ranging from 29 to 246, only 3 studies [34], [38], [40] enrolled ≥100 individuals) were extracted of whom 98 received SE alone and 203 received SE combined with other therapies (151 combined with back school, 12 combined with magnetic field warmer vibration synthetic therapy, 20 combined with intermediate frequency electrotherapy, 10 combined with acupuncture therapy). 46% of the participants included in 8 trials [34], [36]–[42] were men, and sex was not reported for one trial [35]. The mean age of study patients ranged from 20.30 to 49.94 years. Six studies [36]–[41] were done with hospital patients, one [34] with primary care and hospital patients, one [42] with community and hospital patients, and one [35] with university students. Subjects were included according to various inclusion criteria (non-specific LBP, specific LBP or both).

#### SE

The intervention was primarily SE based, and five [38]–[42] of the 9 trials [34]–[42] involved the application of concomitant therapy (eg. back school, 3 [38], [40], [41]/5 [38]–[42]; intermediate frequency electrotherapy, 1 [39]/5 [38]–[42]; acupuncture therapy, 1 [42]/5 [38]–[42]) alongside the intervention and control. Intervention duration ranged from 1 week to 8 weeks with an average length of 5.89 weeks, and intervention frequency was various, ranging from once a week to seven times a week. Eight trials [34], [36]–[42] were done in hospital, and in one [35], the setting was unclear. In 1 study [39], participants spent 20 minutes per session doing SE. In 2 studies [38], [41], participants spent 30 minutes per session doing SE. In 2 studies [34], [36], participants spent 40 minutes per session doing SE. In 1 study [42], participants spent 30 to 60 minutes per session doing SE. And the time spent on the SE per session was unclear in 3 studies [35], [37], [40].

#### Control conditions

Regarding control conditions, one study [37] used a traditional Chinese medical therapy, four studies [34]–[36], [41] used other forms of exercise, two studies [39], [40] used thermomagnetic therapies, and two trials [38], [42] used two separate control interventions, of which one trial [38] used another form of exercise and physical factor therapy combined with drug therapy (meloxicam combined with eperisone hydrochloride tablet) and the remaining one trial [42] used no treatment and a traditional Chinese medical therapy.

#### Outcomes

Outcome measures were self-reported pain (9 trials [34]–[42]), back pain specific functional status (6 trials [34], [36], [38]–[41]), disability (1 trial [42]), skeletal muscle metabolism (1 trial [37]), fear of physical activity and work (1 trial [34]), trunk flexion (1 trial [34]), health care utilization (1 trial [34]), clinical effect (1 trial [39]), anxiety state (1 trial [39]), ability of daily life (1 trial [39]), joint function (1 trial [39]), and trunk extensor strength (1 trial [35]). Of these, the methods used to assess self-reported pain and back pain specific function status among all trials [34]–[42] were different. The pain was measured by VAS (6 [35], [37], [39]–[42]/9 [34]–[42] trials) or NPRS (3 [34], [36], [38]/9 [34]–[42] trials), and back pain specific function status was measured by ODI (4 [36], [39]–[41]/6 [34], [36], [38]–[41] trials) or M-ODI (2 [34], [38]/6 [34], [36], [38]–[41] trials). However, none of the included trials [34]–[42] reported data for return to work, global improvement, health-related quality of life, satisfaction with treatment or adverse events. Data from 9 included studies [34]–[42] were collected at short-term period (9 [34]–[42]/9 [34]–[42] trials), intermediate period (2 [36], [41]/9 [34]–[42] trials) or long-term period (2 [34], [40]/9 [34]–[42] trials). All included trials [34]–[42] reported mean rather than median data for continuous outcomes and reported number of events and total for dichotomous outcomes.

#### Conflict of interest

One study [34] was financed by the Norwegian Fund for Post-Graduate Training in Physiotherapy, and sponsorship of other studies [35]–[42] was not mentioned.

### Methodological Quality of Included Trials

Only 4 trials [34], [38]–[40] adequately reported allocation sequence generation. The remaining 5 trials [35]–[37], [41], [42] were randomized, but no details were provided. One trial [34] had adequate allocation concealment, but we could not determine the allocation concealment in the remaining 8 trials [35]–[42]. None of the 9 included trials [34]–[42] met the blinding of participants, personnel and outcome assessors criterion. Given the direct participant-personnel involvement due to the nature of therapeutic trials, and self-reported outcome measures such as VAS and NPRS, it was not feasible to blind participants, personnel and outcome assessors. While the blinding in 9 included trials [34]–[42] was not feasible, it was still an inherent source of bias that must be highlighted for every trial. The risk of incomplete outcome data was low for only 6 included trials [34]–[37], [39], [42]. Selective outcome reporting was at a low risk of bias in all 9 included trials [34]–[42]. Sources of other bias were unclear in 9 included trials [34]–[42]. Overall, nine included trials [34]–[42] were adjudicated to be of high risk of bias. Table 2 shows the detailed results of the quality assessment of individual characteristics.

**Table 2:** Methodological quality of trials included in systematic review.

Article,Year	Randomsequencegeneration	Allocationconcealment	Blinding ofParticipantsand personnel	Blinding ofoutcomeassessments	Incompleteoutcome data	Selectivereporting	Otherbias	Riskof bias
Gao Baolong (2008)	Unclear	Unclear	High	High	Low	Low	Unclear	High
Unsgaard-Tondel (2010)	Low	Low	High	High	Low	Low	Unclear	High
Guo Xianfeng (2010)	Low	Unclear	High	High	Unclear	Low	Unclear	High
Qin Jiang (2010)	Unclear	Unclear	High	High	Low	Low	Unclear	High
Jin Miao (2011)	Low	Unclear	High	High	Low	Low	Unclear	High
Hu Yuan (2011)	Low	Unclear	High	High	High	Low	Unclear	High
Yoo (2012)	Unclear	Unclear	High	High	Low	Low	Unclear	High
Wang Cong (2012)	Unclear	Unclear	High	High	Unclear	Low	Unclear	High
Liu Pan (2012)	Unclear	Unclear	High	High	Low	Low	Unclear	High

### Effectiveness

#### SE compared with other forms of exercise

**Figure 2:**
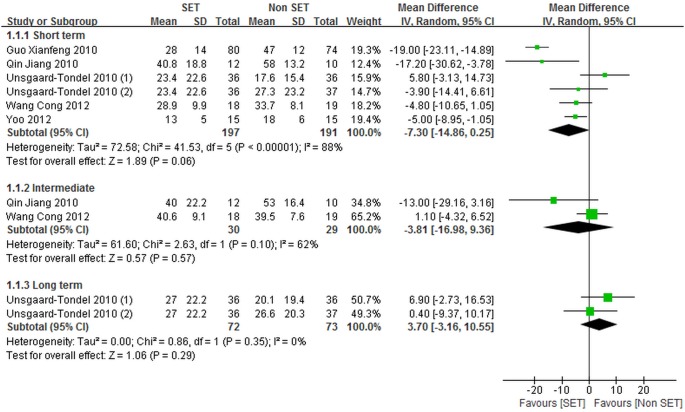
Self-reported pain for sling exercise versus other forms of exercise. Unsgaard-Tondel 2010(1), sling exercise versus motor control exercise; Unsgaard-Tondel 2010(2), sling exercise versus general exercise.

**Figure 3:**
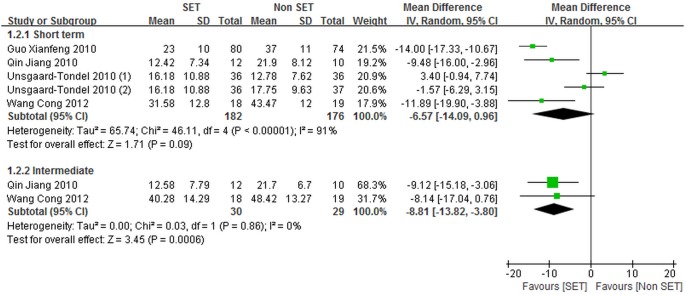
Back pain specific functional status for sling exercise versus other forms of exercise. Unsgaard-Tondel 2010(1), sling exercise versus motor control exercise; Unsgaard-Tondel 2010(2), sling exercise versus general exercise.

Five trials (457 patients) [34]–[36], [38], [41] randomly assigned patients to undergo SE (164 patients) or other forms of exercise (199 patients), and reported data regarding self-reported pain. Among these trials [34]–[36], [38], [41], three [34], [36], [38] evaluated self-reported pain by using NPRS and two trials [35], [41] used VAS. In four studies (427 patients) [34], [36], [38], [41], data regarding back pain specific functional status were reported, in which SE for 149 patients and other forms of exercise for 184 patients were assessed. Among these four studies, two [36], [41] used ODI to measure back pain specific functional status, whereas two [34], [38] utilized M-ODI. Figures 2 and 3 show the overall meta-analysis results of the comparison of SE with other forms of exercise.

The trials [34]–[36], [38], [41] comparing SE with other forms of exercise failed to reveal significant differences in self-reported pain at short-term [–7.30 point (CI = –14.86 to 0.25)], intermediate [–3.81 point (CI = –16.98 to 9.36)], and long-term [3.70 point (CI = –3.16 to 10.55)] follow-up periods.

However, significant heterogeneities for short-term and intermediate self-reported pain were derived when SE was compared with other forms of exercise. The heterogeneity for short-term self-reported pain was substantially high (*I^2^* = 88%). A visual inspection of the forest plot indicated that two studies [36], [38], in which the control treatment was mainly conducted by patients at home, were the most likely source of heterogeneity. A meta-analysis excluding these two studies [36], [38] indicated a substantially reduced heterogeneity (*I* ^2^ = 39%) and revealed a statistically non-significant effect size [–3.01 point (CI = –7.22 to 1.21)]. For intermediate self-reported pain, high *I^2^* value (*I^2^* = 62%) derived in the random effects analysis also suggested a substantial heterogeneity between the included studies [36], [41]. This result may be due to the fact that the included trials [36], [41] differed in patient populations and treatment durations. The baseline scores of pain obtained by Qin et al. [36] were >1.5 points higher than those in Wang et al. [41]; by comparison, the treatment duration presented by Qin et al. [36] was two weeks shorter than that reported by Wang et al. [41]. However, neither of the studies [36], [41] yielded statistically significant effects of SE on the decrease in intermediate self-reported pain compared with other forms of exercise. This result is consistent with that of the pooled analysis.

No significant differences were observed in short-term back pain specific functional status between SE and other forms of exercise [–6.57 point (CI = –14.09 to 0.96)]. We also observed a statistically, but probably not clinically meaningful difference in the intermediate back pain specific functional status [–8.81 point (CI = –13.82 to –3.80)] in favor of SE compared with other forms of exercise.

The statistical heterogeneity for short-term back pain specific functional status was substantially high (*I* ^2^ = 91%). This result may be attributed to the inclusion of one trial [34], in which SE was performed less frequently than in remaining trials [36], [38], [41]. Eliminating this study [34] from the analysis, we no longer observed heterogeneity (*I^2^*  = 0%). Nevertheless, the result was statistically significant, which differed from the conclusion of no statistically significant differences in short-term back pain specific functional status. A subgroup analysis based on the frequency of SE was then performed, and the result showed that smaller effect sizes were achieved in trials, in which SE was performed less frequently.

#### SE compared with traditional chinese medical therapies

**Figure 4:**
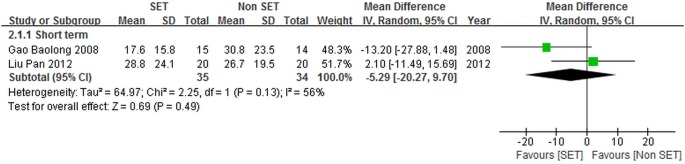
Self-reported pain for sling exercise versus traditional Chinese medical therapies.

Among the included trials [34]–[42], two (89 patients) [37], [42] demonstrated SE and traditional Chinese medical therapies with randomly assigning patients (35 and 34 patients, respectively); the results regarding self-reported pain were determined using VAS. The results of our meta-analysis are shown in Figure 4.

The meta-analysis of two trials [37], [42] showed that SE and traditional Chinese medical therapies were different in terms of the short-term self-reported pain [–5.29 point (CI = –20.27 to 9.70)], but this result was not significant.

The pooled studies [37], [42] demonstrated substantially high levels of heterogeneity (*I* ^2^ = 56%), probably because of the differences between the two included trials [37], [42] in terms of participant populations and treatment durations. The baseline scores of pain observed by Liu et al. [42] were higher than those of Gao et al. [37] by more than two points. By comparison, the treatment duration obtained by Liu et al. [42] was four weeks shorter than that observed by Gao et al. [37]. Neither of the studies [37], [42] indicated the positive effects of SE on the decrease in short-term self-reported pain compared with traditional Chinese medical therapies. This result is similar to that obtained in pooled analysis.

#### SE compared with thermomagnetic therapy

**Figure 5:**
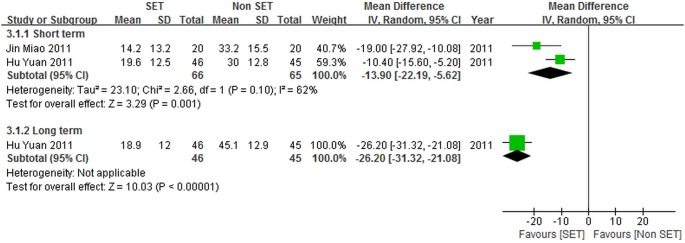
Self-reported pain for sling exercise versus thermomagnetic therapies.

**Figure 6:**
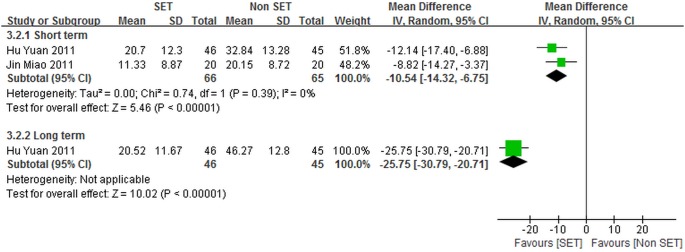
Back pain specific functional status for sling exercise versus thermomagnetic therapies.

Two studies (160 patients) [39], [40] assessed SE performed by 70 patients and thermomagnetic therapy conducted by 70 patients; data on self-reported pain and back pain specific functional status were presented. For the two studies [39], [40], self-reported pain was determined using VAS [39], [40], and both studies [39], [40] assessed back pain specific functional status by using ODI. The weighted mean difference (95% CI) results of the included trials [39], [40] are shown in Figures 5 and 6.

We observed significant differences between SE and thermomagnetic therapy for self-reported pain at either short-term [–13.90 point (CI = –22.19 to –5.62)] or long-term [–26.20 point (CI = –31.32 to –21.08)] follow-up periods.

The heterogeneity of short-term self-reported pain was substantially high (*I* ^2^ = 62%). This result may be due to differences in the frequency of SE between the two included trials [39], [40]. SE was performed less frequently as revealed by Jin et al. [39] than by Hu et al. [40]. The two studies [39], [40] comparing SE with thermomagnetic therapy both reported the positive effects of SE on the reduction of short-term self-reported pain. This result is consistent with that of the pooled analysis.

We recorded both statistically and clinically significant differences between SE and thermomagnetic therapy for back pain specific functional status at either short-term [–10.54 point (CI = –14.32 to –6.75)] or long-term [–25.75 point (CI = –30.79 to –20.71)] follow-up periods.

#### SE compared with the combination of physical agents and drug therapy

Self-reported pain and back pain specific functional status were evaluated in a randomized controlled trial [38], in which 82 patients receiving SE and another 82 patients receiving physical agents combined with drug therapy were included. In this trial [38], self-reported pain was determined using NPRS, and back pain specific functional status was determined using M-ODI.

This trial [38] showed significant differences between SE and physical agents combined with drug therapy for either self-reported pain [–15.00 point (CI = –19.64 to –10.36)] or back pain specific functional status [–10.00 point (CI = –13.70 to –6.30)] at short-term follow-up period.

#### SE combined with acupuncture therapy versus acupuncture therapy alone

One randomized controlled trial [42] with 20 participants receiving acupuncture therapy and another 20 participants receiving acupuncture therapy combined with SE reported data for self-reported pain, which was measured by VAS.

This trial [42] indicated no difference between SE in addition to acupuncture therapy and the acupuncture therapy alone for self-reported pain at short-term follow-up period [–6.30 point (CI = –16.85 to 4.25)].

## Discussion

Systematic reviews [62], [63] have been conducted to evaluate the effects of exercise therapy in participants with chronic LBP. To the best of our knowledge, this study is the first systematic review and meta-analysis that evaluated the effects of SE on chronic LBP management. We derived the following points to provide useful information for caregivers as a guide for patient management, to assist guideline developers in creating guidelines for the use of SE in the chronic LBP treatment, and to allow policy makers in tailoring intervention options towards the desired outcome, available resources, and local healthcare context.

1. The meta-analysis showed no statistically significant or clinically relevant differences in self-reported pain at short-term, intermediate, and long-term follow-up periods between SE and other forms of exercise. SE is no more efficacious than other forms of exercise in improving back pain specific functional status at short-term follow-up period among patients with chronic LBP. However, there is a small, clinically irrelevant benefit of SE over other forms of exercise on intermediate back pain specific functional status, based on an evaluation of less than 60 patients in 2 trials, which both did not perform allocation concealment or blinded outcome assessment, and intention-to-treat analysis was only performed in one of which.
2. SE is as effective as traditional Chinese medical therapies in decreasing short-term self-reported pain, which is consistent with the findings of some published systematic reviews [62], [63] which suggest that exercise therapy and massage are equally effective for chronic LBP.
3. The results indicated that SE was more effective than thermomagnetic therapy for self-reported pain and back pain specific functional status in short- or long-term follow-up periods, but the substantial heterogeneity for short-term self-reported pain comparing SE with thermomagnetic therapy was recorded.
4. In one trial, significant differences between SE and physical agents combined with drug therapy were derived for self-reported pain and back pain specific functional status in the short-tern, which was consistent with the findings of some published trials [64], [65], [66], [67] where exercise therapy is more effective than physical agents or drug therapy in pain relief and functional improvement, and systematic reviews [62], [63] where exercise therapy is more effective than usual care by a general practitioner for chronic LBP.
5. Concerning short-term self-reported pain, no significant differences between SE combined with acupuncture therapy and acupuncture therapy alone were found in one trial.

### Strengths

Our study presented several strengths. We collected data by using a broad search strategy with the following procedures: searching multiple citation databases; soliciting experts for published or unpublished studies; searching trial registries; and screening the references of identified trials. Also, no language restriction was applied. This strategy suggested that our synthesis represented the current state of related studies. We also performed our review according to previously published protocol and methodological guidelines. The consistency of self-reported pain and back pain specific functional status, which were primary outcomes of our review, was satisfactory. All of the studies [34]–[42] in this literature included a measure of self-reported pain and two-thirds [34], [36], [38]–[41] of the studies [34]–[42] included a measure of back pain specific functional status.

### Limitations

First, our systematic review is limited because all of the studies [34]–[42] included in our literature contain methodological drawbacks, which may lead to an overestimated effect. Risk of bias was rated as high in all included trials [34]–[42]. The most frequent methodological shortcoming in included trials [34]–[42] was lack of blinding of patients, caregivers and assessors, due to the nature of the therapeutic interventions indicating that the blinding of patients and caregivers was almost impossible and patient-reported outcomes such as self-reported pain.

Second, the implementation of outcome measures is notably limited. Although the data for self-reported pain and back pain specific functional status were available for the majority of studies [34]–[42], the evaluations of self-reported pain and back pain specific functional status were not standardized, indicating that different trials would use different pain surveys or back pain functional status measures as outcome measures, which could lead to the heterogeneity derived among the trials [34]–[42]. Few researchers reported return to work, global improvement, health-related quality of life, satisfaction with treatment or adverse events; hence, no interpretations on the differences in these outcomes based on the treatment type used were presented. Data for any outcome at intermediate or long-term follow-up period were sparse, suggesting that the efficacy of SE at intermediate and long-term follow-up periods is not well known. Only two of the included studies [36], [41] assessed the intermediate efficacy, and long-term data were only available for one study [34] included in our review.

Third, all included studies [35]–[42], except one study [34] which was sponsored by the Norwegian Fund for Post-Graduate Training in Physiotherapy, did not report sponsoring agent. This serves to limit the study as it could lead to an overestimation of the efficacy of SE in participants with chronic LBP. For example, the inclusion of only trials [35], [36], [38], [41], the sponsorship of which was not mentioned, substantially changed the conclusion of no difference in self-reported pain and back pain specific functional status at short-term follow-up between SE and other forms of exercise.

Fourth, we were unable to retrieve four full-text studies [56], [59]–[61] that may be suitable for the analysis; these studies were then considered to limit the review because whether or not the results of these trials [56], [59]–[61] alter the conclusions derived in this review remains unknown.

Fifth, the sample sizes of trials [34]–[42] included in our systematic review were relatively small, such that only one third [34], [38], [40] of the studies [34]–[42] in our review enrolled 100 or more patients. Several trials [35]–[37], [39], [41], [42] with small sample sizes (<100 participants) are likely to be underpowered to detect relatively small disparities between the effects of SE and that of the control conditions; these studies may overestimate the efficacy of SE in participants with chronic LBP. However, the inclusion of only small trials (<100 participants) [34]–[36], [41] influenced neither the direction nor the significance of the result of our study that no statistically or clinically significant differences in self-reported pain and back pain specific functional status at short-term follow-up between SE and other forms of exercise were derived.

Lastly, the limited number of RCTs [34]–[42] published in this study area might raise the possibility of publication bias. Nine trials [34]–[42] conducted is significantly low in therapeutic research, such that the outcome comparisons included only a few trials [34]–[42], and the results of certain comparisons can easily be dominated by a single trial [38], [40], [42]. No unpublished studies were obtained despite the substantial search.

### Implications for Research

Well-designed and sufficiently powered studies with larger sample sizes should be conducted to clarify whether or not SE improves chronic LBP because of limitations in included trials [34]–[42]. And we identified several implications for further research. First, future trials should adequately use random sequence generation and random concealment, and completely report outcome data. Second, future studies should be conducted in populations that best represent patients with chronic LBP; hence, patient selection should be carefully considered. Third, SE should be well designed, and interventions should be thoroughly described. The treatment affected short-term back pain specific functional status between SE and other forms of exercise at a less extent in trials in which SE was performed less frequently [34]. Hence, studies should evaluate the frequency at which SE is more effective than others for the treatment of chronic LBP. Fourth, the outcome measures for future studies should include pain intensity, functional status, and general health. This recommendation is based on the standard use of outcome measures in back pain research in 1998, including a minimum of pain intensity, functional status, and general health measures [68]. We believe that the intermediate or long-term efficacy associated with SE should be accurately assessed, particularly with standardized methods to report outcomes based on published recommendations. Fifth, further studies should also be conducted regarding the relative cost effectiveness of SE and the potential active mechanisms of SE for chronic LBP. Sixth, researchers should register RCTs and adhere to the CONSORT guidelines to improve the quality of studies. Journals on the field of back pain should adopt reporting guidelines and apply these guidelines in their review process to improve the quality of future reports in this field [51], [69].

## Conclusions

SE is no more efficacious in reducing self-reported pain or improving back pain specific functional status compared with other forms of exercise or traditional Chinese medical therapies with the exception of a statistically significant, but clinically probably irrelevant improvement in intermediate back pain specific functional status between SE and other forms of exercise. In comparison to thermomagnetic therapy or the combination of physical agents and drug therapy, SE more effectively decreased self-reported pain or improved back pain specific functional status. SE in addition to acupuncture therapy is as effective as acupuncture therapy alone for reduction of self-reported pain and improvement of back pain specific functional status. However, the interpretation of our findings is required to be made with caution due to limitations in included trials such as methodological drawbacks and small sample sizes.

## Supporting Information

File S1 **Search strategies for all databases.**
(DOCX)

Checklist S1 **PRISMA 2009 Checklist.**
(DOC)
