# Supplementary material for: Sling Exercise for Chronic Low Back Pain: A Systematic Review and Meta-Analysis
Source: PLoS One. 2014 Jun 11;9(6):e99307. doi: 10.1371/journal.pone.0099307 (PMC4053356; doi:10.1371/journal.pone.0099307)
Supplement: File S1 — Search strategies for all databases. (DOCX) [file pone.0099307.s001.docx]

1. Search Strategy for Cochrane Library

#1 MeSH descriptor: ["random allocation"] explode all trees or MeSH descriptor: [placebos] explode all trees or MeSH descriptor: [single-blind method] explode all trees or MeSH descriptor: [double-blind method] explode all trees or MeSH descriptor: [randomized control trial] explode all trees or MeSH descriptor: [randomized controlled trials as topic] explode all trees or MeSH descriptor: [controlled clinical trials as topic] explode all trees or MeSH descriptor: [controlled clinical trial] explode all trees or MeSH descriptor: [clinical trial] explode all trees or MeSH descriptor: [clinical trials as topic] explode all trees

#2 "random*" or allocation or "random allocation" or placebo or single blind or double blind or "randomized controlled trial*" or RCT or "clinical trial*"

#3 randomized controlled trial:pt or clinical trial:pt

#4 #1 OR #2 OR #3

#5 animals not humans

#6 #4 not #5

#7 back pain:ti,ab,kw or backache:ti,ab,kw or dorsalgia :ti,ab,kw or lumbago:ti,ab,kw or lumbar pain :ti,ab,kw or coccyx :ti,ab,kw or coccydynia :ti,ab,kw or sciatica:ti,ab,kw or spondylosis :ti,ab,kw or back disorder$:ti,ab,kw

#8 sling exercise or exercise therapy

#9 motor control:ti,ab,kw or core stability:ti,ab,kw or stability :ti,ab,kw or stabilization :ti,ab,kw or stabilizing :ti,ab,kw

#10 #8 OR #9

#11 #6 and #7 and #10

2. Search Strategy for Cumulative Index to Nursing and Allied Health Literature

S1 MH("Random Assignment" OR "Placebos" OR "Placebo Effect" OR "Single-Blind Studies" OR "Double-Blind Studies" OR "Triple-Blind Studies" OR "Randomized Controlled Trials" OR "comparative studies" OR "Evaluation Research" OR "Prospective Studies" OR "crossover Design" OR "Prospective Studies" OR "Clinical Trials" OR "Clinical Trial Registry")

S2 TX(random$ OR allocation OR "random allocation" OR placebo$ OR single blind OR double blind OR "randomi?ed controlled trial*" OR "controlled clinical trial*" OR "comparative study" OR "evaluation stud*" OR "follow-up stud*" OR "prospective stud*" OR "cross-over stud*" OR control$ OR prospectiv$ OR volunteer$ OR "RCT" OR "clinical trial*")

S3 PT( randomized controlled trial OR "clinical trial*")

S4 S1 OR S2 OR S3

S5 MH("Back Pain" OR "Low Back Pain")

S6 TX("Back Pain" OR "Low Back Pain" )

S7 TI(backache OR dorsalgia OR lumbago OR "lumbar pain" OR coccyx OR coccydynia OR sciatica OR spondylosis OR "back disorder$")

S8 AB(backache OR dorsalgia OR lumbago OR "lumbar pain" OR coccyx OR coccydynia OR sciatica OR spondylosis OR "back disorder$")

S9 S5 OR S6 OR S7 OR S8

S10 MH(Therapeutic Exercise OR "Exercise Therapy: Muscle Control (Iowa NIC)")

S11 TX(sling exercise OR exercise therapy)

S12 TI(motor control OR TI core stability OR TI stability OR TI stabilization OR TI stabilizing)

S13 AB(motor control OR core stability OR stability OR stabilization OR stabilizing)

S14 S10 OR S11 OR S12 OR S13

S15 S4 AND S9 AND S14

3. Search Strategy for Pubmed

#1 "Single-Blind Method"[Mesh] OR "Double-Blind Method"[Mesh] OR "Randomized Controlled Trials as Topic"[Mesh] OR "Randomized Controlled Trial" [Publication Type] OR "Intention to Treat Analysis"[Mesh] OR "Controlled Clinical Trials as Topic"[Mesh] OR "Clinical Trials as Topic"[Mesh] OR "Clinical Trial" [Publication Type]

#2 "random*"[Text Word] OR allocation[Text Word] OR "random allocation"[Text Word] OR placebo[Text Word] OR single blind[Text Word] OR double blind[Text Word] OR "randomized controlled trial*"[Text Word] OR RCT[Text Word]

#3 randomized controlled trial[Publication Type]

#4 #1 OR #2 OR #3

#5 animals NOT humans

#6 #4 NOT #5

#7 "Back Pain"[Mesh] OR "Low Back Pain"[Mesh]

#8 backache[Title/Abstract] OR dorsalgia[Title/Abstract] OR lumbago[Title/Abstract] OR lumbar pain[Title/Abstract] OR coccyx[Title/Abstract] OR coccydynia[Title/Abstract] OR sciatica[Title/Abstract] OR spondylosis[Title/Abstract] OR back disorder$[Title/Abstract]

#9 #7 OR #8

#10 sling exercise

#11 exercise therapy[Text Word]

#12 motor control[Title/Abstract] OR core stability[Title/Abstract] OR stability[Title/Abstract] OR stabilization[Title/Abstract] OR stabilizing[Title/Abstract]

#13 #10 OR #11 OR #12

#14 #6 AND #9 AND #13

4. Search Strategy for Web of Science

#1 TS=("random*" OR allocation OR "random allocation" OR placebo OR single blind OR single blind method OR double blind OR double blind method OR "randomized controlled trial*" OR "randomised controlled trial*" OR "RCT" OR "clinical trial*")

#2 TS=(back pain OR low back pain or backache OR dorsalgia or lumbago or lumbar pain or coccyx or coccydynia or sciatica or spondylosis or back disorder$)

#3 TS=(sling exercise OR exercise therapy OR motor control OR core stability OR stability OR stabilization or stabilizing)

#4 #1 AND #2 AND #3

Time span=All years. Databases=SCI-EXPANDED, SSCI, A&HCI, CPCI-S, CPCI-SSH.

5. Search Strategy for EMBASE

#1 'randomization'/exp OR 'placebo'/exp OR 'placebo effect'/exp OR 'single blind procedure'/exp OR 'double blind procedure'/exp OR 'randomized controlled trial'/exp OR 'randomized controlled trial (topic)'/exp OR 'controlled clinical trial'/exp OR 'controlled clinical trial (topic)'/exp OR 'clinical trial'/exp OR 'clinical trial (topic)'/exp

#2 random*:ab,ti OR allocation:ab,ti OR "random allocation":ab,ti OR placebo:ab,ti OR single blind:ab,ti OR double blind:ab,ti OR randomised controlled trial*:ab,ti OR randomized controlled trial*:ab,ti OR RCT:ab,ti OR clinical trial*:ab,ti

#3 #1 OR #2

#4 'low back pain'/exp OR 'backache'/exp

#5 backache:ab,ti OR dorsalgia:ab,ti OR lumbago:ab,ti OR lumbar pain :ab,ti OR coccyx :ab,ti OR coccydynia:ab,ti OR sciatica :ab,ti OR spondylosis:ab,ti OR back disorder$:ab,ti

#6 #4 OR #5

#7 sling exercise:ab,ti OR exercise therapy:ab,ti OR motor control:ab,ti OR core stability :ab,ti OR stability :ab,ti OR stabilization:ab,ti OR stabilizing :ab,ti

#8 #3 AND #6 AND #7

6. Search Strategy for Physiotherapy Evidence Database

Abstract & Title: sling exercise

Therapy: no selection

Problem: no selection

Body part: no selection

Subdiscipline: no selection

Topic: no selection

Method: clinical trial

Author/Association: no limited

Title Only: no limited

Source: no limited

Published Since: no limited

New records added since: no limited

Score of at least: no limited

Return: 50(records at a time)

When searching: match all search terms (AND)

1. Search Strategy for Chinese Biomedical Literature Database

#1 "随机分配"[扩展：不加权] OR "对照组"[扩展：不加权] OR "单盲法"[扩展：不加权] OR "双盲法"[扩展：不加权] OR "随机对照试验"[扩展：不加权] OR "临床对照试验"[扩展：不加权] OR "临床试验"[扩展：不加权]

#2 "随机对照试验(主题)"[扩展：不加权] OR "临床对照试验(主题)"[扩展：不加权] OR "临床试验(主题)"[扩展：不加权]

#3 "随机"[全字段：智能] OR "随机分配"[全字段：智能] OR "随机对照"[全字段：智能] OR "对照"[全字段：智能] OR "盲法"[全字段：智能] OR "单盲"[全字段：智能] OR "双盲"[全字段：智能] OR "随机对照试验"[全字段：智能] OR "随机对照试验（文献类型）"[全字段：智能] OR "随机对照研究"[全字段：智能] OR "临床试验"[全字段：智能] OR "临床观察"[全字段：智能] OR "临床研究"[全字段：智能]

#4 (#1) OR (#2) OR (#3)

#5 "腰痛"[扩展：不加权]

#6 "腰痛"[中文标题：智能] OR "下腰痛"[中文标题：智能] OR "腰痛病"[中文标题：智能] OR "腰痛症"[中文标题：智能] OR "下背痛"[中文标题：智能] OR "腰背痛"[中文标题：智能] OR "腰腿疼"[中文标题：智能] OR "腰腿痛"[中文标题：智能] OR "腰腿酸痛"[中文标题：智能] OR "腰椎横突综合征"[中文标题：智能] OR "腰椎滑脱"[中文标题：智能] OR "腰椎滑脱症"[中文标题：智能] OR "腰椎间盘突出"[中文标题：智能] OR "腰椎间盘移位"[中文标题：智能] OR "腰扭伤"[中文标题：智能] OR "强制性脊柱炎"[中文标题：智能] OR "腰筋膜炎"[中文标题：智能] OR "腰肌劳损"[中文标题：智能] OR "腰椎退变"[中文标题：智能] OR "小关节功能紊乱"[中文标题：智能] OR "椎管狭窄"[中文标题：智能] OR "脊柱侧凸"[中文标题：智能]

#7 "腰痛"[摘要：智能] OR "下腰痛"[摘要：智能] OR "腰痛病"[摘要：智能] OR "腰痛症"[摘要：智能] OR "下背痛"[摘要：智能] OR "腰背痛"[摘要：智能] OR "腰腿疼"[摘要：智能] OR "腰腿痛"[摘要：智能] OR "腰腿酸痛"[摘要：智能] OR "腰椎横突综合征"[摘要：智能] OR "腰椎滑脱"[摘要：智能] OR "腰椎滑脱症"[摘要：智能] OR "腰椎间盘突出"[摘要：智能] OR "腰椎间盘移位"[摘要：智能] OR "腰扭伤"[摘要：智能] OR "强制性脊柱炎"[摘要：智能] OR "腰筋膜炎"[摘要：智能] OR "腰肌劳损"[摘要：智能] OR "腰椎退变"[摘要：智能] OR "小关节功能紊乱"[摘要：智能] OR "椎管狭窄"[摘要：智能] OR "脊柱侧凸"[摘要：智能]

#8 "腰痛"[关键词：智能] OR "下腰痛"[关键词：智能] OR "腰痛病"[关键词：智能] OR "腰痛症"[关键词：智能] OR "下背痛"[关键词：智能] OR "腰背痛"[关键词：智能] OR "腰腿疼"[关键词：智能] OR "腰腿痛"[关键词：智能] OR "腰腿酸痛"[关键词：智能] OR "腰椎横突综合征"[关键词：智能] OR "腰椎滑脱"[关键词：智能] OR "腰椎滑脱症"[关键词：智能] OR "腰椎间盘突出"[关键词：智能] OR "腰椎间盘移位"[关键词：智能] OR "腰扭伤"[关键词：智能] OR "强制性脊柱炎"[关键词：智能] OR "腰筋膜炎"[关键词：智能] OR "腰肌劳损"[关键词：智能] OR "腰椎退变"[关键词：智能] OR "小关节功能紊乱"[关键词：智能] OR "椎管狭窄"[关键词：智能] OR "脊柱侧凸"[关键词：智能]

#9 (#5) OR (#6) OR (#7) OR (#8)

#10 "运动疗法"[扩展：不加权]

#11 "吊带"[中文标题：智能] OR "悬吊"[中文标题：智能] OR "运动控制"[中文标题：智能] OR "运动疗法"[中文标题：智能] OR "运动训练"[中文标题：智能] OR "稳定性"[中文标题：智能]

#12 "吊带"[摘要：智能] OR "悬吊"[摘要：智能] OR "运动控制"[摘要：智能] OR "运动疗法"[摘要：智能] OR "运动训练"[摘要：智能] OR "稳定性"[摘要：智能]

#13 "吊带"[关键词：智能] OR "悬吊"[关键词：智能] OR "运动控制"[关键词：智能] OR "运动疗法"[关键词：智能] OR "运动训练"[关键词：智能] OR "稳定性"[关键词：智能]

#14 (#10) OR (#11) OR (#12) OR (#13)

#15 (#4) AND (#9) AND (#14)

1. Search Strategy for Wanfang Database

#1 主题:("随机" + "随机分配" + "随机对照" + "对照" + "盲法" + "单盲" + "双盲" + "随机对照试验" + "临床试验" + "临床观察")

#2 主题:("腰痛" + "下腰痛" + "下背痛" + "腰背痛" + "腰腿疼" + "腰腿痛" + "腰腿酸痛")

#3 主题:("吊带" + "悬吊" + "运动控制" + "运动疗法" + "运动训练" + "稳定性")

#4 (#1) * (#2) * (#3)

1. Search Strategy for China National Knowledge Infrastructure

#1 主题=随机 或者 主题=随机分配 或者 主题=随机对照 或者 主题=对照 或者 主题=盲法 或者 主题=单盲 或者 主题=双盲 或者 主题=随机对照试验 或者 主题=随机对照研究 或者 主题=临床试验 或者 主题=临床观察 或者 主题=临床研究（精确匹配）

#2 主题=腰痛 或者 主题=下腰痛 或者 主题=下背痛 或者 主题=腰背痛 或者 主题=腰腿疼 或者 主题=腰腿痛 或者 主题=腰腿酸痛 或者 主题=腰椎 或者 主题=腰扭伤 或者 主题=脊柱 或者 主题=腰筋膜炎 或者 主题=腰肌劳损 或者 主题=小关节功能紊乱 或者 主题=椎管狭窄 （精确匹配）

#3 主题=吊带 或者 主题= 悬吊 或者 主题= 运动控制 或者 主题= 运动疗法 或者 主题= 运动训练 或者 主题= 稳定性（精确匹配）

#4 #1 并且 #2 并且 #3
